# Location‐Scale Meta‐Analysis and Meta‐Regression as a Tool to Capture Large‐Scale Changes in Biological and Methodological Heterogeneity: A Spotlight on Heteroscedasticity

**DOI:** 10.1111/gcb.70204

**Published:** 2025-05-02

**Authors:** Shinichi Nakagawa, Ayumi Mizuno, Kyle Morrison, Lorenzo Ricolfi, Coralie Williams, Szymon M. Drobniak, Malgorzata Lagisz, Yefeng Yang

**Affiliations:** ^1^ Department of Biological Sciences, Faculty of Science University of Alberta Edmonton Canada; ^2^ Evolution and Ecology Research Centre, School of Biological, Earth and Environmental Sciences University of New South Wales Sydney New South Wales Australia; ^3^ School of Mathematics and Statistics The University of New South Wales Sydney Australia; ^4^ Institute of Environmental Sciences, Faculty of Biology Jagiellonian University Kraków Poland

**Keywords:** Bayesian statistics, double‐hierarchical model, generalized linear mixed‐effects model, multilevel meta‐analysis, phylogenetic meta‐analysis

## Abstract

Heterogeneity is a defining feature of ecological and evolutionary meta‐analyses. While conventional meta‐analysis and meta‐regression methods acknowledge heterogeneity in effect sizes, they typically assume this heterogeneity is constant across studies and levels of moderators (i.e., homoscedasticity). This assumption could mask potentially informative patterns in the data. Here, we introduce and develop a location‐scale meta‐analysis and meta‐regression framework that models both the mean (location) and variance (scale) of effect sizes. Such a framework explicitly accommodates heteroscedasticity (differences in variance), thereby revealing when and why heterogeneity itself changes. This capability, we argue, is crucial for understanding responses to global environmental change, where complex, context‐dependent processes may shape both the average magnitude and the variability of biological responses. For example, differences in study design, measurement protocols, environmental factors, or even evolutionary history can lead to systematic shifts in variance. By incorporating hierarchical (multilevel) structures and phylogenetic relationships, location‐scale models can disentangle the contributions from different levels to both location and scale parts. We further attempt to extend the concepts of relative heterogeneity and publication bias into the scale part of meta‐regression. With these methodological advances, we can identify patterns and processes that remain obscured under the constant variance assumption, thereby enhancing the biological interpretability and practical relevance of meta‐analytic results. Notably, almost all published ecological and evolutionary meta‐analytic data can be re‐analysed using our proposed analytic framework to gain new insights. Altogether, location‐scale meta‐analysis and meta‐regression provide a rich and holistic lens through which to view and interpret the intricate tapestry woven with ecological and evolutionary data. The proposed approach, thus, ultimately leads to more informed and context‐specific conclusions about environmental changes and their impacts.

## Graphical abstract

Meta‐analyses are widely used to understand how species and ecosystems respond to global change, but most assume that variability among studies is constant. This assumption can hide important biological patterns related to resilience, sensitivity, or inconsistency in responses. Here, we highlight how location‐scale meta‐analysis can model both average effects and differences in variability across studies. This approach uncovers richer insights into ecological and evolutionary responses to global change, helping researchers better predict not only the direction but also the stability of biological changes across different conditions.

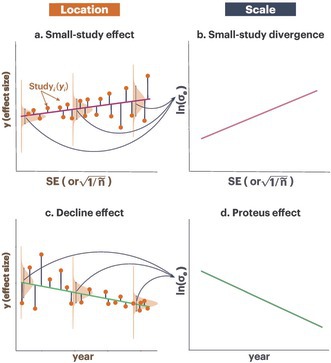

## 1 Introduction

Meta‐analysis has become an indispensable tool in ecology and evolutionary biology; it offers a means to synthesize results across diverse studies and to detect broad‐scale patterns and biases (e.g., publication bias) that may be invisible at the level of individual investigations (Gurevitch et al. 2018; Nakagawa et al. 2017; Yang et al. 2022). Yet, the process of explaining heterogeneous datasets is fraught with challenges. Studies differ not only in their focal taxa, systems, and conditions but also in methodologies, measurement protocols, and analytical approaches. Such complexity leads to substantial heterogeneity in effect sizes, which could obscure underlying biological signals and hinder our understanding of global ecological change. Indeed, variation not due to sample size differences across studies frequently accounts for more than 90% (i.e., $I^{2}>0.9$) in ecological and evolutionary meta‐analyses (Senior et al. 2016; but see Yang, Noble et al. 2023; note that in medicine, $I^{2}>0.75$ is considered to be high; Higgins et al. 2003).

Conventional meta‐analytic frameworks attempt to accommodate heterogeneity by introducing random effects and moderator variables. These approaches recognize that effect sizes are not identical and that moderators—such as climate gradients, habitat types, taxonomic groups, or methodological factors—may help explain some of the variance (Gurevitch et al. 2018; Nakagawa, Yang, et al. 2023; Nakagawa et al. 2017). However, linear models including standard meta‐analysis and meta‐regressions typically assume homoscedasticity, meaning that the variance of effect sizes remains constant across levels of these moderators (Viechtbauer and López‐López 2022). Such an assumption can be unrealistic, as both biological processes and methodological variation often influence not only the magnitude but also the variability of responses (Cleasby and Nakagawa 2011). For example, under some environmental conditions, species or communities may display highly consistent responses, while in others, responses may be much more variable. Similarly, one type of measurement can be more consistent than another type of measurement.

**FIGURE 1:**
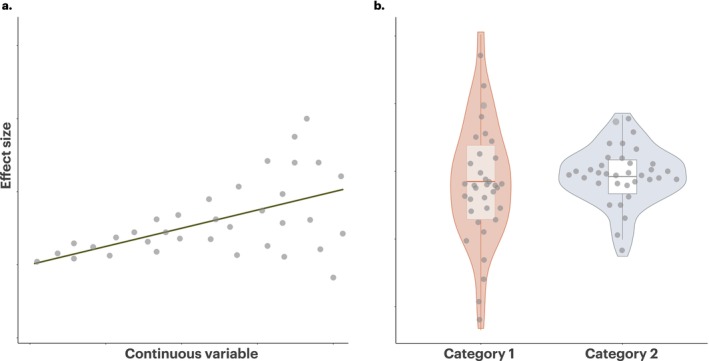
Visualizing heteroscedasticity: (a) an example of a continuous moderator (e.g., temperature, elevation, or sampling effort) with variance in effect sizes increasing as moderator values increase. (b) an example of a categorical moderator (e.g., females vs. males or Method A vs. Method B) with Category 1 having more variation in effect sizes than Category 2.

In environmental sciences, including global change biology, this distinction between average responses and their variability is crucial. Understanding how variance patterns shift along environmental gradients or across study designs can illuminate processes of adaptation, resilience, or sensitivity (Pecl et al. 2017; Urban 2015). For instance, certain anthropogenic changes, such as climate warming or habitat fragmentation, might not only alter the mean response of organisms but also produce more divergent responses among studies due to underlying differences in selection regimes, resource availability, or measurement uncertainty (Figure 1; e.g., Pottier et al. 2022; Mathot et al. 2024). Without explicitly modeling the variance as a function of moderators, these subtle but important patterns of variability remain hidden (Cleasby and Nakagawa 2011; Nakagawa et al. 2015; Senior et al. 2020).

Recent advances in statistical modeling offer solutions to this problem. Location‐scale (mean–variance) modeling frameworks have been long recognized in other areas of statistics and quantitative genetics (Lee and Nelder 1996, 2006; Rönnegård et al. 2010; Sae‐Lim et al. 2015) and more recently, they have been adopted in ecology, evolution, and environmental sciences (Cleasby et al. 2015; Mulder et al. 2016; O'Dea et al. 2022; Pitt et al. 2020). However, their application to meta‐analysis, a domain inherently characterized by high‐level heterogeneity, remains under‐explored (Viechtbauer and López‐López 2022). We can directly model heteroscedasticity and partition the drivers of variability more explicitly by extending the concept of meta‐regression to include both location (mean) and scale parts (variance). Moreover, multilevel and phylogenetic extensions of location‐scale models allow researchers to capture hierarchical structures and evolutionary histories that shape both the average effect sizes and their variation (dispersion).

In this paper, we present location‐scale meta‐analysis and meta‐regression as a flexible, broadly applicable methodology for analysing ecological and evolutionary meta‐analytic data (cf., Blowes 2024). We outline the theoretical foundation of the approach and illustrate how to incorporate moderators into both the mean and variance components. We then show how the framework can be adopted to accommodate multilevel and phylogenetic models. Additionally, we describe how the idea of heterogeneity in meta‐analysis can be extended in the scale part and how regression‐based methods can be expanded to test new types of publication biases in the scale part. We provide illustrative examples of model implementations to demonstrate the usefulness and insights that can be gained, with three different ecological datasets using statistical software, R (R Core Team 2024; for an online tutorial, see link). Finally, we discuss how our proposed methodology improves our understanding of global change biology and potentially better predicts the future impact of global changes by revealing patterns of variability that mirror complex ecological and evolutionary realities.

## 2 Theory

Below, we develop location‐scale meta‐analytic models of increasing complexity in five steps. These steps include extending the quantification of heterogeneity and the detection of publication bias from the mean part to the scale part (cf., Viechtbauer and López‐López 2022).

### 2.1 Random‐Effects Meta‐Analysis and Meta‐Regression

The starting point of most ecological and evolutionary meta‐analyses is the random‐effects model (Nakagawa and Santos 2012). Consider a set of studies indexed by i=1,…,K, each reporting an effect size $y_{i}$ (i.e., one effect size per study). The random‐effects model can be written as (Hedges 1983):

(1) $$y_{i}=\beta_{0}+e_{i}+m_{i}$$

where $\beta_{0}$ is the overall meta‐analytic mean (intercept), $e_{i}$ represents the study effect for i‐th study (also, the i‐th effect‐size effect under this example, as the number of effect sizes and studies are the same), and $m_{i}$ is the sampling error of the effect size estimate. Typically, we assume:

(2) $$e_{i}∼N\left(0,\sigma_{e}^{2}\right)$$

and

(3) $$m_{i}∼N\left(0,\sigma_{m_{i}}^{2}\right)$$

where $\sigma_{e}^{2}$ is the between‐study (between‐effect‐size) variance (in the literature, often noted as $\tau^{2}$), and $\sigma_{m_{i}}^{2}$ is sampling variance for i‐th study (effect size) assumed to be known (often computed as a plug‐in estimator from study‐level sample sizes or other data). For example, when the effect size is a Fisher's z‐transformed correlation coefficient $z_{r}$, the sampling variance often takes a simple form like $1/\left(n_{i}-3\right)$, where $n_{i}$ is the sample size of the i‐th study.

Note that the random‐effects model assumes different studies have different means (Hedges 1983); if there is no (between‐study) heterogeneity or $\sigma_{e}^{2}=0$, then the random‐effects model reduces to the fixed‐effect model where the overall mean ($\beta_{0}$) is the “true” mean for all the studies. Also, note that $\sigma_{e}^{2}$ is hard to interpret as a general measure of heterogeneity because its magnitude depends on what type of effect size one uses. Therefore, the most common and relative measure of heterogeneity in meta‐analysis (see Yang, Noble et al. 2023) is:

(4) $$I^{2}=\frac{\sigma_{e}^{2}}{\sigma_{e}^{2}+{\bar{\sigma^{2}}}_{m}}$$

with

(5) $${\bar{\sigma^{2}}}_{m}=\frac{\sum \frac{1}{\sigma_{m_{i}}^{2}}\;\left(K-1\right)}{{\left(\sum \frac{1}{\sigma_{m_{i}}^{2}}\right)}^{2}\;-\sum {\left(\frac{1}{\sigma_{m_{i}}^{2}}\right)}^{2}}$$

where ${\bar{\sigma^{2}}}_{m}$ is a typical (average) sampling variance (Higgins and Thompson 2002; Higgins et al. 2003); note that to obtain $I^{2}$ or related indices, we use estimated parameters, i.e., variance components (e.g., via restricted maximum likelihood, REML, estimator or Bayesian estimators using Markov Chain Monte Carlo, MCMC). In this form, (between‐study) heterogeneity is expressed as a ratio in relation to the total variance (i.e., $\sigma_{e}^{2}+{\bar{\sigma^{2}}}_{m}$; we extend the idea of heterogeneity in meta‐analysis in Sections 2.3 and 2.4 below).

When moderators are introduced (e.g., $x_{1i},x_{2i},\ldots ,x_{\mathrm{pi}}$; i.e., having p moderators), the model extends to:

(6) $$y_{i}=\beta_{0}+\beta_{1}x_{1i}+\cdots +\beta_{p}x_{\mathrm{pi}}+e_{i}+m_{i}.$$

This standard meta‐regression framework allows one to examine how moderators (covariates) influence the average effect size. However, it still maintains the assumption of a constant heterogeneity variance $\sigma_{e}^{2}$, ignoring potential differences in variance structure among different levels or values of the moderators. Note that a moderator (or predictor; x) can be a continuous or categorical variable.

We note that when a categorical moderator has h levels, we have h−1 dummy variables (i.e., (h−1) xs) and corresponding regression coefficients $\beta_{s}$ are usually contrasts (differences) between a reference category (level) and another category (level). A recent survey shows that almost all ecological and evolutionary meta‐regression analyses had at least one categorical moderator (97%) while only around 30% of meta‐regression analyses included at least one continuous moderator (Nakagawa, Lagisz, et al. 2023). This finding indicates that dummy variables are very common in meta‐regression analyses in ecology and evolution.

Given extremely high heterogeneities in ecological and evolutionary meta‐analyses (Gurevitch et al. 2018), it is notable that meta‐regression models, which include moderators (Equation 6), are the main analytical focus rather than meta‐analytic models (i.e., intercept‐only models; Equation 1). A significant moderator in a meta‐regression is a piece of “synthesis‐generated evidence” because such evidence cannot be identified by examining each separate study (Cooper 2015; Nakagawa et al. 2017).

### 2.2 Random‐Effects Location‐Scale Meta‐Regression

Location‐scale meta‐regression explicitly models not only the location (mean) of the effects but also their scale (variance), allowing heteroscedasticity to be a function of moderators (cf., Cleasby and Nakagawa 2011). We extend the above meta‐regression (Equation 6) by decomposing the model into a location part and a scale part (Viechtbauer and López‐López 2022):

(7) $$y_{i}=\beta_{0}^{\left(l\right)}+\beta_{1}^{\left(l\right)}x_{1i}+\cdots +\beta_{p}^{\left(l\right)}x_{\mathrm{pi}}+e_{i}^{\left(l\right)}+m_{i}^{\left(l\right)}$$

with

(8) $$e_{i}^{\left(l\right)}∼N\left(0,\sigma_{e_{i}}^{2}\right)$$

where $\beta_{p}^{\left(l\right)}$ are location parameters (i.e., affecting the mean part), and we allow the residual variance $\sigma_{e_{i}\left(l\right)}^{2}$ to vary by modeling the logarithm of its squared‐root (i.e., standard deviation $\ln\left(\sigma_{e_{i}}\right)$) as a linear function of moderators:

(9) $$\ln\left(\sigma_{e_{i}}\right)=\beta_{0}^{\left(s\right)}+\beta_{1}^{\left(s\right)}x_{1i}+\cdots +\beta_{p}^{\left(s\right)}x_{\mathrm{pi}}$$

where $\beta_{p}^{\left(s\right)}$ coefficients indicate how much moderators influence heterogeneity itself.

In the scale part, any factor $x_{\mathrm{pi}}$ influencing the scale part $\left(\beta_{p}^{\left(s\right)}\neq 0\right)$ implies that heterogeneity itself changes systematically with the moderator (i.e., heteroscedasticity). For example, a binary (categorical) moderator might predict distinct variances between two different groups (e.g., aquatic organisms having higher variance than terrestrial counterparts as in Example 1 below). Both the logarithm of the variance $\ln\left(\sigma_{e_{i}}^{2}\right)$ or standard deviation $\ln\left(\sigma_{e_{i}}\right)$ can be the response variable in the scale part and the choice is a matter of preference; for example, O'Dea et al. (2022) uses variance or $\ln\left(\sigma_{e_{i}}^{2}\right)$ while Cleasby et al. (2015) uses standard deviation $\ln\left(\sigma_{e_{i}}\right)$. We use $\ln\left(\sigma_{e_{i}}\right)$ because our choice of implementation, the R package brms (Bürkner 2017), uses standard deviation rather than variance. We should also note that a set of moderators does not need to be the same in the location and scale parts. Yet, without any clear prior predictions, one could start with the same moderators in both parts.

### 2.3 Multilevel Meta‐Analysis and Multilevel Location‐Scale Meta‐Regression

Many meta‐analyses contain hierarchical structures, such as multiple effect sizes nested within studies (cf., Rodriguez et al. 2023; Williams et al. 2021). Indeed, a survey revealed that such a nested structure was present in 73 out of 73 meta‐analytic studies (100%) in environmental sciences (Nakagawa, Yang, et al. 2023). Before introducing the multilevel location‐scale meta‐regression, we briefly review the standard multilevel meta‐analytic model, which can be written as:

(10) $$y_{i}=\beta_{0}+u_{j\left[i\right]}+e_{i}+m_{i}$$

with

(11) $$u_{j}∼N\left(0,\sigma_{u}^{2}\right)$$

where $u_{j\left[i\right]}$ is the between‐study effect for the j‐th study (or of the i‐th effect size) and $\sigma_{u}$ is the between‐study variance and $e_{i}$ follows Equation (2), but it is notable that $\sigma_{e_{i}}^{2}$ is now the within‐study variance (effect‐size‐level variance). As mentioned earlier, the between‐study variance is often noted as $\tau^{2}$ in the meta‐analytic literature. We use $\sigma^{2}$ for all variance notations (e.g., $\sigma_{u}^{2}$, $\sigma_{e}^{2}$, $\sigma_{m}^{2}$).

Notably, $m_{i}$ can be distributed following Equation (3), but it is more likely to take the following form:

(12) $$m_{i}∼N\left(0,V\right)$$

where V is a block diagonal matrix capturing the sampling covariance structure within and among effect sizes from the same study. For example, if we have 20 studies and, then, we have 20 blocks and, say, we can see the first 3 studies where they have 3, 1, and 2 effect sizes, respectively. Let us further assume that the first 2 effect sizes in study 1 are derived from the same subjects and so are the two effect sizes in study 3 (elsewhere, we called such types of dependencies as correlated sampling errors to distinguish this dependence from another type of dependence due to belonging to the same studies, controlled by the random effect $u_{j\left[i\right]}$; see Nakagawa, Yang, et al. 2023; Yang, Macleod, et al. 2023). We can now write the first three blocks of V as (note that the boxes are drawn to show three blocks, which corresponds to three studies):

(13) V1−3=σm12ρmσm1σm20ρmσm2σm1σm22000σm32000σm42000σm52ρmσm5σm6ρmσm6σm5σm62

where $\rho_{m}$ is correlation between sampling variances (e.g., of effect size 1 and 2; $\sigma_{m_{1}}^{2}$ and $\sigma_{m_{2}}^{2}$); the value of $\rho_{m}$ takes a value between 0 and 1 yet an exact value is unknown apart from some special conditions (e.g., effect size 1 and 2 shared a control group or we have access to original data so that we can sometimes obtain $\rho_{m}$ directly; see Noble et al. 2017). Therefore, we often assume that either $\rho_{m}=0.5$ or 0.8 (Noble et al. 2017; Pustejovsky and Tipton 2022); note the variance–covariance matrix V can be easily constructed by, for example, the function vcalc in the R package metafor (Viechtbauer 2010). Alternatively, robust variance estimators can be employed; this approach offers flexibility in handling complex dependency structures among sampling errors, as we do not need to define the value of $\rho_{m}$. Interestingly, Pustejovsky and Tipton (2022) recommend the combined use of V and robust variance estimators (see also Hedges et al. 2010).

By extending the concept of the relative heterogeneity above (Equation 4), we can now define three types of $I^{2}$ (Nakagawa and Santos 2012; Nakagawa, Yang, et al. 2023; Yang, Noble, et al. 2023):

(14) $$I_{B}^{2}=\frac{\sigma_{u}^{2}}{\sigma_{u}^{2}+\sigma_{e}^{2}+{\bar{\sigma^{2}}}_{m}}$$

(15) $$I_{W}^{2}=\frac{\sigma_{e}^{2}}{\sigma_{u}^{2}+\sigma_{e}^{2}+{\bar{\sigma^{2}}}_{m}}$$

and

(16) $$I_{T}^{2}=\frac{\sigma_{u}^{2}+\sigma_{e}^{2}}{\sigma_{u}^{2}+\sigma_{e}^{2}+{\bar{\sigma^{2}}}_{m}}.$$

As one can see, $I_{B}^{2}$ is the relative heterogeneity of between‐study effects (differences), while $I_{W}^{2}$ is that of within‐study effects, and the sum of these two is $I_{T}^{2}$ (total relative heterogeneity; for details and other types of relative heterogeneity measures, see Yang, Noble et al. 2023).

Now we can define a multilevel location‐scale meta‐regression model building upon the multilevel meta‐analytic model (Equation 10); the location part is:

(17) $$y_{i}=\beta_{0}^{\left(l\right)}+\beta_{1}^{\left(l\right)}x_{1i}+\cdots +\beta_{p}^{\left(l\right)}x_{\mathrm{pi}}+u_{j\left[i\right]}^{\left(l\right)}+e_{i}^{\left(l\right)}+m_{i}^{\left(l\right)}$$

with

(18) $$u_{j}^{\left(l\right)}∼N\left(0,\sigma_{u\left(l\right)}^{2}\right)$$

where $u_{j\left[i\right]}^{\left(l\right)}$ is the between‐study effect for the j‐th study that i‐th effect size belongs to, $\sigma_{u\left(l\right)}^{2}$ is the between‐study variance, and other symbols as above. The scale equation can be the same as Equation (9). Yet, it is important to notice that we could add the between‐study effect to the scale part:

(19) $$\ln\left(\sigma_{e_{i}}\right)=\beta_{0}^{\left(s\right)}+\beta_{1}^{\left(s\right)}x_{1i}+\cdots +\beta_{p}^{\left(s\right)}x_{\mathrm{pi}}+u_{j\left[i\right]}^{\left(s\right)}.$$

By including the random effects (between‐study effects) in both the location and scale equations and correlating them, we can model scenarios where studies with larger (or smaller) mean effects might also tend to exhibit greater (or smaller) variance; note that models with random effects in both location and scale parts are known as “double‐hierarchical” models (Lee and Nelder 1996, 2006). Formally, we can define a bivariate normal distribution for the between‐study effects:

(20) $$\left(\begin{array}{c} u_{j}^{\left(l\right)}\\ u_{j}^{\left(s\right)} \end{array}\right)∼N\left(\left(\begin{array}{c} 0\\ 0 \end{array}\right),\left(\begin{array}{cc} \sigma_{u\left(l\right)}^{2} & \rho_{u}\sigma_{u\left(l\right)}\sigma_{u\left(s\right)}\\ \rho_{u}\sigma_{u\left(l\right)}\sigma_{u\left(s\right)} & \sigma_{u\left(s\right)}^{2} \end{array}\right)\right).$$

**FIGURE 2:**
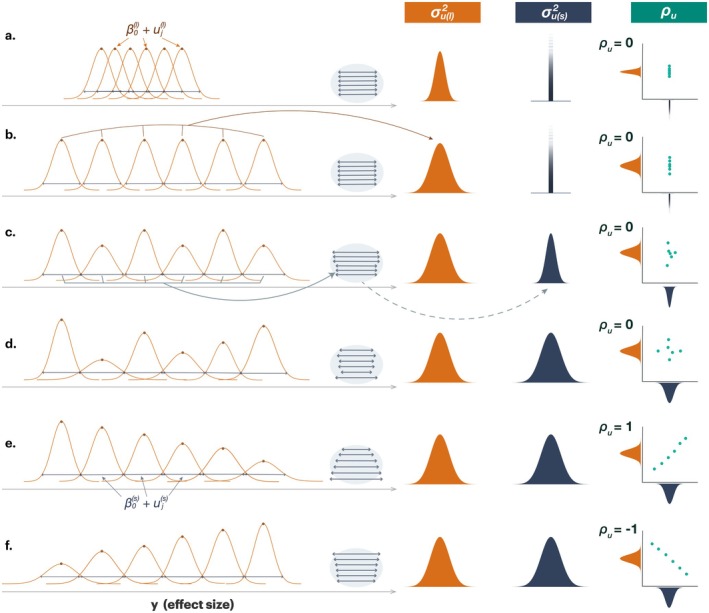
Illustration of location‐scale models with different combinations of random effects in the location and scale parts. (a) Depicts a scenario where each study (orange curves) has its mean (i.e., $\beta_{0}^{\left(l\right)}+u_{j}^{\left(l\right)}$) and variance (i.e., $\beta_{0}^{\left(s\right)}+u_{j}^{\left(s\right)}$). Between‐study variation in the average effect size is represented by $\sigma_{u\left(l\right)}^{2}$ (light‐orange distribution), and between‐study variation in variance is represented by $\sigma_{u\left(s\right)}^{2}$ (navy distribution), which is zero (no variation or homoscedasticity). Correlation between these two random effects ($\rho_{u}$) can be zero, positive, or negative, leading to different patterns (in this case, zero), (b) in this scenario, everything is the same apart from between‐study variance in means are larger than scenario a, (c) in this case, there are variations in variation, (d) in this case, each study differs in mean and variance with $\rho_{u}=0$. (e) a positive correlation, $\rho_{u}=1$, means that higher mean effects co‐occur with greater variance. (f) a negative correlation, $\rho_{u}=-1$, means that higher mean effects co‐occur with lower variance. Each panel on the right shows a schematic distribution of $u_{j}^{\left(l\right)}$ (orange) and $u_{j}^{\left(s\right)}$ (blue), along with their correlation in a scatterplot. These scenarios highlight how location‐scale approaches can capture diverse patterns of heterogeneity.

Here, the value of $\sigma_{u\left(s\right)}^{2}$ indicates the magnitude of differences in variance between studies (also a large value indicates a likely existence of heteroscedasticity; Figure 2), and $\rho_{u}$ measures the correlation between the location and scale random effects, and unlike $\rho_{m}$ in an earlier section, $\rho_{u}$ spans between −1 and 1 (not between 0 and 1). Under normal circumstances, we do not expect any correlation between $u_{j}^{\left(l\right)}$ and $u_{j}^{\left(s\right)}$ because the default assumption is that mean and variance are independent in normally (Gaussian) distributed data (Figure 2 showing different patterns of this correlation). Yet in biology, mean and variance may often be positively correlated, which is known as Taylor's law (Taylor 1961; see also Nakagawa et al. 2015). Of relevance, researchers have found that there is a positive correlation between sampling variance ($\sigma_{m_{i}}^{2}$) and heterogeneity (of means), equivalent measures of $\sigma_{u\left(s\right)}^{2}$ (Figure 2f); that is, primary studies with smaller sample sizes tend to have larger heterogeneity (or larger residual value or $\ln\left(\sigma_{e_{i}}\right)$) (IntHout et al. 2015; Stanley et al. 2022). Given small studies often have large effect sizes in magnitudes, this finding indicates that we may find that larger effects in magnitude are related to high variance in a meta‐analysis (i.e., nonzero $\rho_{u}$ between effect sizes and heterogeneity), a pattern that may suggest selection bias or other methodological artefacts (e.g., smaller studies reporting both inflated means and noisier/variable results; Stanley et al. 2022). Larger studies are less likely to be affected by these issues, and thus, large‐study divergence is unlikely to occur as mentioned earlier.

Notably, adding the between‐study (random) effect in the scale part results in two extra parameters to estimate, i.e., $\sigma_{u\left(s\right)}^{2}$ and $\rho_{u}$; in addition to j between‐study effects $u_{i}^{\left(s\right)}$, which naturally requires more data. Therefore, such a location‐scale meta‐regression model with the between‐study effect in the scale part may require larger meta‐analytic datasets (for more discussion, see our examples below). Nevertheless, it could be informative to estimate $\sigma_{u\left(s\right)}^{2}$ and $\rho_{u}$ regardless of dataset size. Therefore, we suggest before fitting a multilevel location‐scale meta‐regression, we can first fit the following meta‐analytic model:

(21) $$y_{i}=\beta_{0}^{\left(l\right)}+u_{j\left[i\right]}^{\left(l\right)}+e_{i}^{\left(l\right)}+m_{i}^{\left(l\right)}$$

and

(22) $$\ln\left(\sigma_{e_{i}}\right)=\beta_{0}^{\left(s\right)}+u_{j\left[i\right]}^{\left(s\right)}.$$

This meta‐analysis provides a more accurate error estimate of the overall effect (i.e., $\beta_{0}$) when there exists nonnegligible variation in variance. We propose that this meta‐analytic model should be the starting point if one is to investigate heteroscedasticity. This is because nonzero $\sigma_{u\left(s\right)}^{2}$ warrants location‐scale meta‐regression in the same way as heterogeneity in a normal meta‐analytic model calls for a (standard) meta‐regression analysis (Nakagawa and Santos 2012).

Additionally, in this location‐scale meta‐analytic model (Equations ((21), (22))), both $\sigma_{u\left(s\right)}^{2}$ and $\rho_{u}$ can be estimated as in Equation (20). Yet, in location‐scale models with the between‐study effects in both parts (i.e., double‐hierarchical models), it is possible not to model $\rho_{u}$ by assuming $\rho_{u}=0$ as below, especially, when modeling $\rho_{u}$ leads to difficulties in model convergence (which could help convergence and mixing in a Bayesian model; see the examples below and the online tutorial):

(23) $$\left(\begin{array}{c} u_{j}^{\left(l\right)}\\ u_{j}^{\left(s\right)} \end{array}\right)∼N\left(\left(\begin{array}{c} 0\\ 0 \end{array}\right),\left(\begin{array}{cc} \sigma_{u\left(l\right)}^{2} & 0\\ 0 & \sigma_{u\left(s\right)}^{2} \end{array}\right)\right).$$

It is interesting and maybe insightful to compare these two kinds of heterogeneity: (1) heterogeneity in mean and (2) heterogeneity in variance. Yet, we cannot compare these two parameters directly because they are on different scales (i.e., the latter is on the log‐normal scale). In the next section, we resolve this very issue.

### 2.4 Extending the Idea of Heterogeneity to Location‐Scale Models

Earlier, we introduced the relative measure of heterogeneity, $I^{2}$ (variance‐standardized measure). For Equation (21) (the location part), we can also calculate three types of $I^{2}$ as with Equations ((14), (15), (16)). For example, $I_{B}^{2}$ can be obtained as:

(24) $$I_{B}^{2}=\frac{\sigma_{u\left(l\right)}^{2}}{\sigma_{u\left(l\right)}^{2}+{\bar{\sigma^{2}}}_{e}+{\bar{\sigma^{2}}}_{m}}$$

with

(25) $${\bar{\sigma^{2}}}_{e}=\exp\left(2\beta_{0}^{\left(s\right)}+2\sigma_{u\left(s\right)}^{2}\right)$$

where $\beta_{0}^{\left(s\right)}$ is from Equation (22) and $\sigma_{u\left(s\right)}^{2}$ is the variance component for the between‐study effects $u_{j\left[i\right]}^{\left(s\right)}$ from the same equation (O'Dea et al. 2022). Yet, it is not possible to extend $I^{2}$ to the scale part as the part lacks an equivalent of the sampling error variance (i.e., $\sigma_{m_{i}}^{2}$). Although less used, there is an alternative measurement of relative heterogeneity for meta‐analysis, which is mean‐standardized (Cairns and Prendergast 2022). Using the random‐effects model (Equation 1), we can define this measure (${\mathrm{CV}}_{H}$) as (Takkouche et al. 1999):

(26) $${\mathrm{CV}}_{H}=\frac{\sigma_{e}}{|\beta_{0}|}$$

where CV denotes the coefficient of variation, and $|\beta_{0}|$ is the absolute values of the overall mean (they match $|\beta_{0}|$ in Equation (1) but not necessarily in meta‐regression models).

For a multi‐level meta‐analysis (Equation 10), we have (Yang, Noble et al. 2023):

(27) $${\mathrm{CV}}_{H\left(B\right)}=\frac{\sigma_{u}}{|\beta_{0}|}$$

(28) $${\mathrm{CV}}_{H\left(W\right)}=\frac{\sigma_{e}}{|\beta_{0}|}$$

and

(29) $${\mathrm{CV}}_{H\left(T\right)}=\frac{\sqrt{\sigma_{e}^{2}+\sigma_{u}^{2}}}{|\beta_{0}|}$$

where ${\mathrm{CV}}_{H\left(B\right)}$, ${\mathrm{CV}}_{H\left(W\right)}$, and ${\mathrm{CV}}_{H\left(T\right)}$ are between‐study, within‐study and total relative heterogeneity although ${\mathrm{CV}}_{H\left(B\right)}+{\mathrm{CV}}_{H\left(W\right)}\neq {\mathrm{CV}}_{H\left(T\right)}$ (but ${\mathrm{CV}}_{H\left(B\right)}^{2}+{\mathrm{CV}}_{H\left(W\right)}^{2}={\mathrm{CV}}_{H\left(T\right)}^{2}$; Yang, Noble et al. 2023; cf., Equations (14), (15), (16)).

Mentioned earlier, the location‐scale meta‐analytic model in the previous section (Equations (21), (22)) has the between‐study effects in both the location and scale part. We can, therefore, define relative heterogeneity (${\mathrm{CV}}_{H}$) for both the location and scale parts, using Equations ((21), (22)):

(30) $${\mathrm{CV}}_{H\left(B\right)}^{\left(l\right)}=\frac{\sigma_{u\left(l\right)}}{|\beta_{0}|}$$

and

(31) $${\mathrm{CV}}_{H\left(B\right)}^{\left(s\right)}=\sqrt{\exp\left(\sigma_{u\left(s\right)}^{2}\right)-1}$$

where ${\mathrm{CV}}_{H\left(B\right)}^{\left(l\right)}$ and ${\mathrm{CV}}_{H\left(B\right)}^{\left(s\right)}$ are between‐study relative heterogeneity for the location and scale part, respectively note that Equation (31) is CV for standard deviation as we model standard deviation; if we want to make it CV for variance, we need to use $4\sigma_{u\left(s\right)}^{2}$ instead of $\sigma_{u\left(s\right)}^{2}$ in Equation (31). Although Equation (31) does not look like a coefficient of variation, it indeed is (see Cleasby et al. 2015; O'Dea et al. 2022). These two types of CV can be comparable in theory (yet note that these measures were originally developed for ratio scale variables, which have zero as the minimum value). For example, if both CVs are similar values, variability in mean and variance is similar (see Figure 2). We note that these measures have yet to be used in meta‐analyses, so it is hard to gauge their usefulness (cf., Yang, Noble et al. 2023). Yet, the consistency of studies in terms of mean and variance should be of importance for many meta‐analysts.

### 2.5 Modeling Four Types of Publication Bias in Location‐Scale Models

Publication biases, such as small‐study effects and decline effects, can influence meta‐analytic results (Rothstein et al. 2005). The small‐study effect happens when selective publications of small studies with only significant effects bias the overall mean. The decline effect occurs when larger and statistically significant effects are published earlier than smaller and nonstatistically significant effects, resulting in a decline in the magnitude of the overall effect over time (also known as time‐lag bias; Koricheva and Kulinskaya 2019); while an incline effect may theoretically be possible, practically, it is rarely, if ever, observed (Yang, Lagisz, et al. 2023). Indeed, both types of publication bias are common in ecology and evolution (Yang, Sánchez‐Tójar, et al. 2023; Yang et al. 2022). One of the notable strengths of meta‐analysis is its ability to detect such publication biases.

For example, small‐study effects can be examined by regressing $y_{i}$ on the square root of sampling variance (standard error, se; Egger et al. 1997; Moreno et al. 2009).

(32) $$y_{i}=\beta_{0}+\beta_{1}{\mathrm{se}}_{i}+\cdots +u_{j\left[i\right]}+e_{i}+m_{i}$$

where ${\mathrm{se}}_{i}$ is sampling standard deviation for i‐th effect size (the square root of sampling variance, also often referred to as sampling standard error; for Zr, it $1/\left(n_{i}-3\right)$). Alternatively, we can use $\sqrt{1/{\tilde{n}}_{i}}$, where ${\tilde{n}}_{i}$ is an effective sample size for i‐th effect size and the use of such effective sample size avoids known correlation between effect size point estimates and their standard error as in standardized mean difference, SMD (more often referred to as Cohen's d or Hedges' g; see Nakagawa et al. 2022):

(33) $$y_{i}=\beta_{0}+\beta_{1}\sqrt{1/{\tilde{n}}_{i}}+\cdots +u_{j\left[i\right]}+e_{i}+m_{i}$$

Without the presence of a small‐study effect (publication bias), there should be no relationship between effect sizes and ${\mathrm{se}}_{i}$ (or $\sqrt{1/{\tilde{n}}_{i}}$), which form a funnel shape by effect size values converging to an overall value as ${\mathrm{se}}_{i}$ (or $\sqrt{1/{\tilde{n}}_{i}}$) decreases. If $\beta_{1}\neq 0$, this suggests funnel asymmetry and hence the small‐study effect. A funnel asymmetry could happen due to other moderators than the effective sample size. Therefore, it is important to model other moderators, which account for variation in the data.

Similarly, the decline effect can be examined by including a centered publication year $c\left({\text{year}}_{i}\right)$ as a moderator (note that centring is not essential, yet helps interpretation; see Schielzeth 2010):

(34) $$y_{i}=\beta_{0}+\beta_{1}c\left({\text{year}}_{i}\right)+u_{j\left[i\right]}+\cdots +e_{i}+m_{i}$$

By combining these moderators (${\mathrm{se}}_{i}/\sqrt{1/{\tilde{n}}_{i}}$ and $c\left({\text{year}}_{i}\right)$), we can model both location and scale to detect how biases affect not only average effect sizes but also their heterogeneity. The location‐scale version might look like (cf., Viechtbauer and López‐López 2022):

(35) $$y_{i}=\beta_{0}^{\left(l\right)}+\beta_{1}^{\left(l\right)}\sqrt{1/{\tilde{n}}_{i}}+\beta_{2}^{\left(l\right)}c\left({\text{year}}_{i}\right)+\cdots +u_{j\left[i\right]}^{\left(l\right)}+e_{i}^{\left(l\right)}+m_{i}^{\left(l\right)}$$

and

(36) $$\ln\left(\sigma_{e_{i}}\right)=\beta_{0}^{\left(s\right)}+\beta_{1}^{\left(s\right)}\sqrt{1/{\tilde{n}}_{i}}+\beta_{2}^{\left(s\right)}c\left({\text{year}}_{i}\right)+\cdots .$$

If $\beta_{1}^{\left(s\right)}$ is statistically significant, it implies that heterogeneity increases with decreasing sample size (often linked to small‐study effects; IntHout et al. 2015; Viechtbauer and López‐López 2022), whereas a significant $\beta_{2}^{\left(s\right)}$ might indicate a “Proteus” effect, where variance (heterogeneity) in effect sizes decline over time (Trikalinos and Ioannidis 2005). The reason for the Proteus effect is that it is easier to publish papers that contradict the initial findings, which leads to high variance, yet, over time, variance in effect sizes declines as a consensus emerges (Trikalinos and Ioannidis 2005). However, in ecology and evolution, we predict that heterogeneity can increase over time because an initial finding in one population (or one species) is often tested in more populations (and more species), increasing variability in effect sizes over time. This is the opposite of what the original Proteus effect meant, expanding what a Proteus effect means to any changes in effect sizes over time.

**FIGURE 3:**
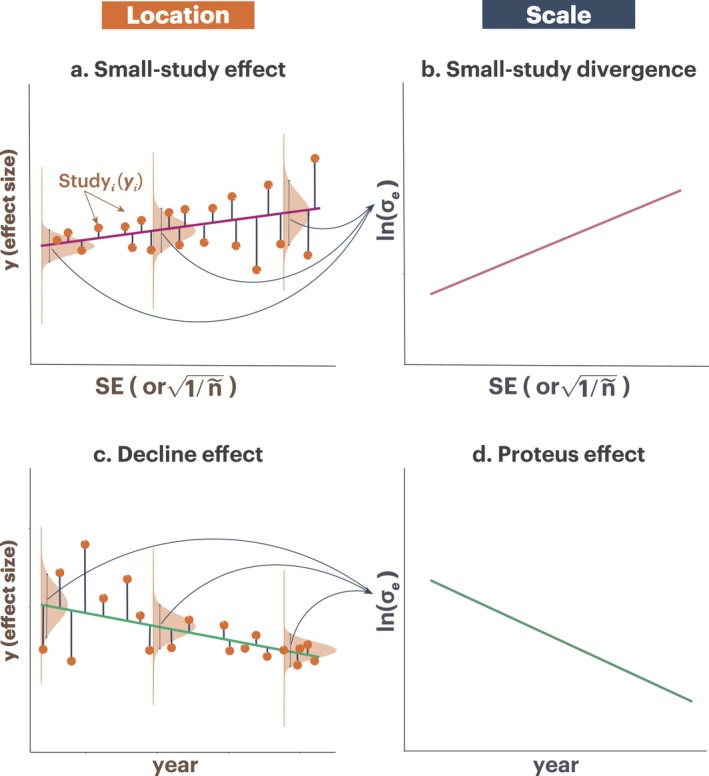
Four types of publication bias: (a) *Small‐study effect (location part)*: A conventional Egger‐type test regresses observed effect sizes (y) on their standard errors (SE or $\sqrt{1/\tilde{n}}$). A significant slope suggests that smaller (less precise) studies yield systematically different mean effects. (b) *Small‐study divergence (scale part)*: Location‐scale models allow testing whether less precise (smaller) studies exhibit not just different average outcomes but also greater (or lesser) variance. (c) *Decline effect (location part)*: Also called the time‐lag bias, where earlier studies may report inflated effects that gradually decline over publication years (green slope). (d) *Proteus effect (scale part)*: Over time, variance among effect sizes could increase or decrease. A decrease might reflect an emerging consensus, whereas an increase may arise if subsequent studies expand across different conditions, species, or methodologies. By including moderators such as sample size or publication year in the scale component, location‐scale models can detect biases that inflate variance, revealing more complex patterns of publication distortions beyond mean shifts alone (note all effect sizes are assumed to be independent so one effect size per study).

Therefore, using Equation ((35), (36)), we can quantify: (a) a small‐study effect (the location part; Figure 3a), (b) a decline effect (the location part; Figure 3b), (c) a small‐study effect on variance, which we name “small‐study divergence” (it could be “small‐study” convergence, but it is unlikely see below; the scale part; Figure 3c), and (d) a Proteus effect (the scale part; Figure 3d). Such comprehensive examinations have not been tried but can be valuable for diagnosing publication biases in meta‐analytic data.

### 2.6 Phylogenetic (Multilevel) Location‐Scale Meta‐Analysis and Meta‐Regression

Ecological and evolutionary meta‐analyses often deal with species‐level data, where evolutionary history can shape both the mean and variance of effect sizes (Cinar et al. 2022; Hadfield and Nakagawa 2010; Nakagawa and Santos 2012). By building upon the multilevel model (Equation 10), a phylogenetic multilevel meta‐analytic model can be written as:

(37) $$y_{i}=\beta_{0}+a_{k\left[i\right]}+s_{k\left[i\right]}+u_{j\left[i\right]}+e_{i}+m_{i}$$

with

(38) $$a_{k}^{\left(l\right)}∼N\left(0,\sigma_{a\left(l\right)}^{2}A\right)$$

and

(39) $$s_{k}^{\left(l\right)}∼N\left(0,\sigma_{s\left(l\right)}^{2}\right)$$

where $a_{k\left[i\right]}^{\left(l\right)}$ captures the phylogenetic effect for the k‐th species, and $s_{k\left[i\right]}^{\left(l\right)}$ is the nonphylogenetic (species‐level random) effect for the k‐th species, each of them is normally distributed with $\sigma_{a\left(l\right)}^{2}A$ and $\sigma_{s\left(l\right)}^{2}$ and A is a correlation matrix containing relatedness of k species (Cinar et al. 2022; Hadfield and Nakagawa 2010; Nakagawa and Santos 2012). It is notable that the ratio between $\sigma_{a\left(l\right)}^{2}$ and $\sigma_{s\left(l\right)}^{2}$ can quantify the relative strength of “phylogenetic signal” in a dataset. It is known either as λ or phylogenetic heritability ($H^{2}$; Lynch 1991; Cinar et al. 2022; but see Pearse et al. 2023;):

(40) $$\lambda =H^{2}=\frac{\sigma_{a}^{2}}{\sigma_{a}^{2}+\sigma_{s}^{2}}$$

Based on the above, a phylogenetic location‐scale meta‐regression model can be written as (cf. Halliwell 2025; Nakagawa et al. 2025):

(41) $$y_{i}=\beta_{0}^{\left(l\right)}+\beta_{1}^{\left(l\right)}x_{1i}+\cdots +\beta_{p}^{\left(l\right)}x_{\mathrm{pi}}+a_{k\left[i\right]}^{\left(l\right)}+s_{k\left[i\right]}^{\left(l\right)}+u_{j\left[i\right]}^{\left(l\right)}+e_{i}^{\left(l\right)}+m_{i}^{\left(l\right)}$$

and the scale component can similarly incorporate moderators:

(42) $$\ln\left(\sigma_{e_{i}}\right)=\beta_{0}^{\left(s\right)}+\beta_{1}^{\left(s\right)}x_{1i}+\cdots +\beta_{p}^{\left(s\right)}x_{\mathrm{pi}}+\cdots .$$

By doing so, we can test whether certain clades or evolutionary lineages exhibit inherently different levels of heterogeneity by species‐level moderators (e.g., two different species groups according to their taxonomy). This phylogenetic extension helps to unravel how evolutionary history, along with environmental or methodological moderators, shapes both the magnitude and dispersion of ecological and evolutionary responses. Note that we could add the between‐study effect ($u_{i}^{\left(s\right)}$) in the scale part. Also, it is possible even to incorporate the phylogenetic and nonphylogenetic effects ($a_{k\left[i\right]}^{\left(s\right)}$ and $s_{k\left[i\right]}^{\left(s\right)}$) in the scale part, but we would not recommend such models unless one has a relatively large dataset; the more complex the model, the more data points are required (cf., Cinar et al. 2022). Also, the location part of a meta‐analytic model can be written as:

(43) $$y_{i}=\beta_{0}^{\left(l\right)}+a_{k\left[i\right]}^{\left(l\right)}+s_{k\left[i\right]}^{\left(l\right)}+u_{i}^{\left(l\right)}+e_{i}^{\left(l\right)}+m_{i}^{\left(l\right)}$$

with the scale part being Equation (22); such a phylogenetic multilevel location‐scale meta‐analytic model can be run before fitting a meta‐regression counterpart.

## 3 Worked Examples

Here, we provide illustrative examples by re‐analysing data from three published meta‐analyses. Our aim here is to show examples of models we described above, and, therefore, we note that our model structure (e.g., the absence of phylogenetic relatedness) and the choice of moderators are unlikely to be biologically or methodologically the best given these datasets. That is, our examples may present models that could be too simplistic and fail to fully capture the complexities of these datasets. For implementation, we primarily use the R package, brms (Bürkner 2017) but for some models, we also use metafor (Viechtbauer 2010) and blsmeta (Rodriguez et al. 2023; Williams et al. 2021); note that results from all three packages brms, metafor, and blsmeta are all consistent with each other. The full R scripts, along with the datasets, are available on our tutorial page, which can also serve as an introduction to fitting standard meta‐analysis and meta‐regression using these packages. Notably, in the online tutorial (link), we start each example fitting a multilevel location‐scale meta‐analytic model, which we have recommended above as a starting point of modeling (i.e., Equations 21 and 22). Below, however, we focus on results from location‐scale meta‐regression models, mainly using Equations (17) and (9) rather than Equations (17) and (19); this is because the former mix and converge more easily and also multiple R packages can fit this model, although the latter can be a better model in some cases (note that it is possible to decide which model is better using Bayesian model selection using, for example, Widely Applicable Information Criterion, WAIC or leave‐one‐out cross‐validation, loo‐cv; Vehtari et al. 2017; see also Blowes 2024).

### 3.1 Example 1: Biological and Methodological Categorical Moderators

**FIGURE 4:**
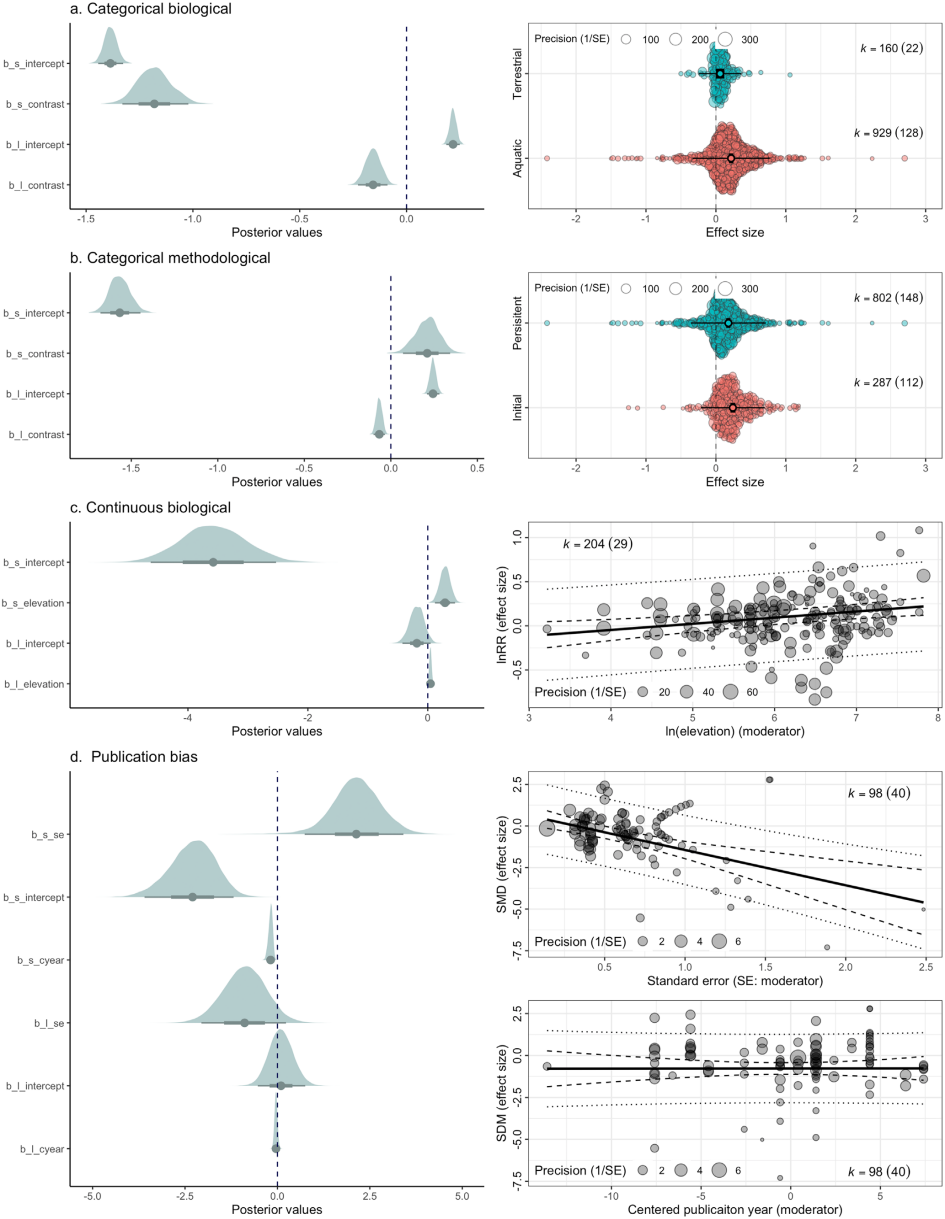
Illustrative location‐scale meta‐regression examples covering categorical, continuous, and publication bias moderators using “ggplot2” (Wickham 2011), “tidybayes” (Kay 2020) and “orchaRd” packages (Nakagawa, Lagisz, et al. 2023). (a) *Categorical biological* moderator: contrasting terrestrial (blue) versus aquatic (red) organisms (terrestrial‐aquatic). The left panel shows posterior distributions of four key parameters from the Bayesian location‐scale model: the intercept (b_l_intercept), the habitat contrast (b_l_contrast) in the location part, and the corresponding intercept (b_s_intercept) and habitat contrast (b_s_contrast) in the scale part. The vertical dashed line indicates zero, aiding the interpretation of effect direction with thick lines showing 66% credible intervals and thin whiskers 95% credible intervals. The right panel or orchard plot depicts effect sizes by habitat (vertical axis) and their average precision (bubble size) or sampling effort (horizontal jitter), illustrating that aquatic organisms showed not only larger mean effect sizes but also higher variance with thick lines showing 95% confidence intervals and thin whiskers 95% prediction intervals. (b) *Categorical methodological* moderator: initial versus persistent temperature exposures (persistent‐initial). The left panel similarly displays the posterior distributions for intercept and contrast in both location and scale parts, revealing that persistent exposures yield higher variance than initial exposures. The right panel shows an orchard plot of effect sizes by the method category, with bubble size again proportional to precision (1/SE). (c) *Continuous biological* moderator (e.g., log‐elevation). The left panel highlights how both the location (e.g., b_l_elevation) and scale (b_s_elevation) slopes differ from zero, indicating that mean effect sizes increase with elevation while variance likewise expands. The right panel shows a scatter of effect sizes across the moderator axis (ln(elevation)), with bubble sizes proportional to precision, along with the fitted location trend (solid line) and its 95% confidence intervals (dashed lines) and 95% prediction intervals (dotted lines). (d) *Publication‐bias* variables: sample size (SE) and publication year (cyear). On the left, the location part (b_l_se, b_l_cyear) tests for the small‐study effect and decline effect (no statistical evidence for these effects), while the scale part (b_s_se, b_s_cyear) examines small‐study divergence and the Proteus effect (evidence for these effects). The right panels illustrate partial regressions against standard error and centered publication year, each bubble sized by precision, with the fitted lines in black and 95% intervals in dashed lines. The bubble plot, based on a standard meta‐regression not location‐scale meta‐regression, for se showed a small study effect; yet, this effect was not detected in the corresponding location‐scale model. This indicates the small‐study divergence (which was not modeled) has created the small‐study effect in the normal meta‐regression, emphasizing the importance of all four publication biases as proposed here.

Pottier et al. (2022) studied the capacity of animals to increase thermal tolerance via heat exposure (increased temperature) using a meta‐analysis with the ratio of acclimation response between control and heat‐exposed groups, as effect sizes. Using multilevel location‐scale meta‐regression (i.e., Equations 17 and 9), we re‐analysed their dataset, whether habitat (living aquatic [aqu.] vs. terrestrial [ter.] habitat) and “method” (experiments testing either early/initial exposure [ini.] or persistent exposure [per.]) moderate not only the mean effect but also variances. Indeed, not only terrestrial organisms had significantly lower heat tolerance overall than aquatic counterparts, overall ($\beta_{\left[\mathrm{ter}.-\mathrm{aqu}.\right]}^{\left(l\right)}$: −0.16, 95% CI: −0.23 to −0.29) but also they had significantly lower variability ($\beta_{\left[\mathrm{ter}.-\mathrm{aqu}.\right]}^{\left(s\right)}$: −1.18, 95% CI: −1.33 to −1.02; Figure 4a). Also, persistent exposures, overall, increased heat tolerance yet, significantly less than early (initial) exposure ($\beta_{\left[\mathrm{per}.-\mathrm{ini}.\right]}^{\left(l\right)}$: −0.07, 95% CI: −0.10 to −0.03), although persistent exposures generated significantly more variability ($\beta_{\left[\mathrm{per}.-\mathrm{ini}.\right]}^{\left(s\right)}$: 0.21, 95% CI: 0.07 to 0.34; Figure 4b). These reanalyses highlight the often neglected roles of biological and methodological moderators in meta‐analyses; we expect and predict heteroscedasticity (i.e., significant contrasts (slopes) on the scale part ($\beta^{\left(s\right)}$) are prevalent in ecological and evolutionary meta‐analytic data).

### 3.2 Example 2: A Continuous Biological Moderator

Midolo et al. (2019) examined how plant traits change along a relevant gradient, using log response ratio (lnRR; comparing trait differences over differences in elevatoin). Here, we re‐analysed one of the traits, nitrogen concentration per unit of area (N*area*), using a location‐scale meta‐regression model. As with the original authors, we found an increase in elevation difference accompanied by a significant increase in N*area* ($\beta_{\left[\text{elevation}\right]}^{\left(l\right)}$: 0.05, 95% CI: 0.01 to 0.08). More importantly, variances among effect sizes (lnRR for N*area*) also increased as the elevation increased ($\beta_{\left[\text{elevation}\right]}^{\left(s\right)}$: 0.29, 95% CI: 0.12 to 0.46); (Figure 4c). Although continuous moderators like elevation here are less common in meta‐analytic data sets (Nakagawa, Lagisz, et al. 2023), heteroscedasticity in such moderators may be more common than we assume (i.e., homoscedasticity).

### 3.3 Example 3: Modeling Publication Bias in the Location and Scale Part

Neuschulz et al. (2016) studied the effect of forest disturbance on pollination, seed dispersal, seed predation, recruitment and herbivory during plant regeneration, using a meta‐analysis with standardized means difference (SMD) as their effect size. We used their data set to test the four types of publication biases described above by fitting sampling standard error (se; note the higher the standard error, usually, the smaller the sample size) and the centered publication year (cyear). Although we found little statistical evidence for the small‐study effect and the decline effect ($\beta_{\left[\mathrm{se}\right]}^{\left(l\right)}$: −0.89, 95% CI: −2.06 to 0.23); ($\beta_{\left[\text{cyear}\right]}^{\left(l\right)}$: −0.04, 95% CI: −0.12 to 0.04), we found such evidence for small‐study divergence ($\beta_{\left[\mathrm{se}\right]}^{\left(s\right)}$: −0.19, 95% CI: 0.31 to −0.09) as well as the Proteus effect with variance going down over time ($\beta_{\left[\text{cyear}\right]}^{\left(s\right)}$: 2.13, 95% CI: 0.74 to 3.41; Figure 4d). This example points out that the current practice of just testing for the small study and the decline effect may miss the complexity of publication bias, missing the important insights gained by testing publication bias on the scale part, i.e., the small‐study divergence and Proteus effect.

## 4 Discussion

In this paper, we have introduced (phylogenetic) multilevel location‐scale meta‐analysis and meta‐regression as a new methodological advance to better capture, understand, and interpret heterogeneity and heteroscedasticity in ecological and evolutionary meta‐analyses with illustrative examples from global change biology (cf., Viechtbauer and López‐López 2022; Blowes 2024). By jointly modeling the location (mean) and scale (variance) of effect sizes, this approach surpasses conventional frameworks that treat variance as a single, homogeneous quantity. Below, we highlight the key advantages and implications of this framework in eight points.

First, the location‐scale framework enhances biological interpretability. Variability in responses is not merely noise; it can reflect underlying ecological and evolutionary processes. When variance differs systematically across moderators, we understand whether certain environments, taxa, or conditions channel responses into restricted or variable outcomes. Such insights are highly relevant in a rapidly changing world, where both shifting averages and expanding or contracting variances across populations may signal adaptive capacity, vulnerability, or underlying ecological complexity (Pecl et al. 2017; Urban 2015). Notably, such changes in variation in response can be easily visualized by orchard plots (for categorical variables) or bubble plots (for continuous variables; Nakagawa et al. 2021; Nakagawa, Lagisz, et al. 2023; Nakagawa, Yang, et al. 2023) (see Figure 4).

Second, location‐scale modeling helps disentangle methodological sources of heterogeneity. Differences in study design, measurement techniques, or analytical choices may inflate the variance of reported effect sizes (cf., Dougherty and Shuker 2015; Christie et al. 2019; Mathot et al. 2024). Incorporating methodological moderators into the scale component allows us to identify when and how systematic sources of variability arise, guiding future research toward more consistent protocols and improving the overall reliability and comparability of meta‐analytic findings (Blowes 2024).

Third, related to the first two points, we can also inform predictions, for example, under global change scenarios. As environmental drivers intensify, understanding not just how mean responses shift but also how variance itself changes is critical. Increased variability may indicate an ecological opportunity for some species or impending instability for others. Modeling changes in variance gives us an additional tool to anticipate the directions and magnitudes of uncertainty that will accompany shifts in mean responses, ultimately improving our ability to forecast and manage biological responses to global change.

Fourth, integrating hierarchical (multilevel) structures into location‐scale models accommodates ecological and evolutionary meta‐analytic datasets with multiple effect sizes per study (cf., Viechtbauer and López‐López 2022). This approach not only provides a clearer picture of the relative contributions of study‐level and effect‐level factors but also elucidates between‐study heterogeneity in the scale part as well as in the location part (Yang, Noble et al. 2023). Indeed, we have proposed a multilevel location‐scale meta‐analytic model with the between‐study effects in both parts as the starting point for exploring heterogeneity in mean and variance (e.g., comparing ${\mathrm{CV}}_{H\left(B\right)}^{\left(l\right)}$ and ${\mathrm{CV}}_{H\left(B\right)}^{\left(s\right)}$).

Fifth, incorporating phylogenetic structures into location‐scale models not only controls for nuisance nonindependence but also deepens our evolutionary understanding (Cinar et al. 2022; Hadfield and Nakagawa 2010; Nakagawa and Santos 2012). By accounting for shared ancestry, we can determine whether specific clades inherently produce more variable responses, possibly due to broader genetic diversity, greater plasticity, or more complex ecological interactions. Phylogenetic extensions allow us to identify evolutionary patterns in both mean effect sizes and their variability.

Sixth, the location‐scale framework enables more comprehensive investigations of publication biases; we have outlined the four types of publication biases (the small‐study effect, decline effect, small‐study divergence, and Proteus effect). Traditional tests focus on detecting biases in mean effect sizes (Koricheva and Kulinskaya 2019; Nakagawa et al. 2022). By including moderators in the scale component, we can also examine biases in heterogeneity itself. For instance, we may identify when small studies or more recent publications not only inflate mean effects but also increase variance, revealing previously undetected dimensions of bias. Such multifaceted examinations of publication biases can improve the robustness and trustworthiness of meta‐analytical conclusions.

Seventh, therefore, the multifaceted approach enhances the interpretability of meta‐analytic findings for stakeholders and policymakers (Koricheva and Kulinskaya 2019; Yang, Noble et al. 2023). Rather than presenting a single mean effect size with a uniform measure of heterogeneity, we can specify when and where heterogeneity increases or decreases. These more detailed insights can guide resource allocation, monitoring efforts, and mitigation strategies for conditions associated with the greatest uncertainties or susceptibilities.

Eighth, more broadly, location‐scale meta‐analytic models present an opportunity for synthesis and comparability across a wide range of ecological and evolutionary contexts. By applying this method to various research questions, we can begin to build a general understanding of how heterogeneity responds to both biological and methodological factors (cf., Cleasby and Nakagawa 2011). This holistic approach promises to enrich our grasp of biodiversity, ecosystem functioning, and evolutionary potential as they unfold under changing environmental conditions. Importantly, given reasonable sample sizes (e.g., 40 effect sizes; indicated by simulation in Rodriguez et al. 2023; see also Blázquez‐Rincón et al. 2025), all published ecological and evolutionary meta‐analyses can be re‐analysed with our proposed models to investigate heteroscedasticity.

In summary, location‐scale meta‐analysis and meta‐regression models, with multilevel, phylogenetic, and publication‐bias extensions, provide a versatile and biologically interpretable framework for meta‐analysis. They allow researchers to understand how moderators influence average effect sizes and reveal the conditions under which heterogeneity is amplified or diminished. This yields deeper ecological and evolutionary insights, refines our interpretations of meta‐analytic results, and ultimately advances our understanding of complex biological responses to global environmental change.

## Author Contributions

**Shinichi Nakagawa:** conceptualization, formal analysis, funding acquisition, investigation, methodology, project administration, resources, supervision, visualization, writing – original draft, writing – review and editing. **Yefeng Yang:** conceptualization, formal analysis, investigation, methodology, software, validation, writing – review and editing. **Malgorzata Lagisz:** investigation, visualization, writing – review and editing. **Szymon M. Drobniak:** conceptualization, writing – review and editing. **Kyle Morrison:** investigation, writing – review and editing. **Ayumi Mizuno:** investigation, visualization, writing – review and editing. **Coralie Williams:** investigation, writing – review and editing. **Lorenzo Ricolfi:** investigation, writing – review and editing.

## Conflicts of Interest

The authors declare no conflicts of interest.

## Data Availability

All relevant data and code are available at: https://doi.org/10.5281/zenodo.15079661.
